# Crystal structure, Hirshfeld surface analysis, DFT and mol­ecular docking investigation of 2-(2-oxo-1,3-oxazolidin-3-yl)ethyl 2-[2-(2-oxo-1,3-oxazolidin-3-yl)eth­oxy]quinoline-4-carboxyl­ate

**DOI:** 10.1107/S2056989020015960

**Published:** 2021-01-01

**Authors:** Younos Bouzian, Cemile Baydere, Necmi Dege, Noureddine Hamou Ahabchane, Joel T. Mague, Abdulmalik Abudunia, Khalid Karrouchi, El Mokhtar Essassi

**Affiliations:** a Laboratory of Heterocyclic Organic Chemistry URAC 21, Pole of Competence, Pharmacochemistry, Av Ibn Battouta, BP 1014, Faculty of Sciences, Mohammed V, University, Rabat, Morocco; b Department of Physics, Faculty of Arts and Sciences, Ondokuz Mayıs University, 55139-Samsun, Turkey; c Department of Chemistry, Tulane University, New Orleans, LA 70118, USA; d Department of Pharmacology, Faculty of Clinical Pharmacy, University of Medical and Applied Sciences, Yemen; e Laboratory of analytical Chemistry and Bromatology, Faculty of Medicine and Pharmacy, Mohammed V University, Rabat, Morocco

**Keywords:** crystal structure, Covid-19, DFT, Hirshfeld surface analysis, oxazolidine, quinoline, Mol­ecular docking

## Abstract

In the mol­ecular structure of the title compound, C_20_H_21_N_3_O_7_, the quinoline ring system is slightly bent, with a dihedral angle between the phenyl and the pyridine rings of 3.47 (7)°. In the crystal, corrugated layers of mol­ecules extending along the *ab* plane are generated by C—H⋯O hydrogen bonds. The inter­molecular inter­actions were qu­anti­fied by Hirshfeld surface analysis and two-dimensional fingerprint plots. The most significant contributions to the crystal packing are from H⋯H (42.3%), H⋯O/O⋯H (34.5%) and H⋯C/ C⋯H (17.6%) contacts. Mol­ecular orbital calculations providing electron-density plots of the HOMO and LUMO as well as mol­ecular electrostatic potentials (MEP) were computed, both with the DFT/B3LYP/6–311 G++(d,p) basis set. A mol­ecular docking study between the title mol­ecule and the COVID-19 main protease (PDB ID: 6LU7) was performed, showing that it is a good agent because of its affinity and ability to adhere to the active sites of the protein.

## Abstract 2

In the crystal of the title compound, corrugated layers of mol­ecules extending along the *ab* plane are generated by C—H⋯O hydrogen bonds.

## Chemical context   

Quinoline and its derivatives have attracted the inter­est of synthetic and biological chemists because of their inter­esting chemical and pharmacological properties (Chu *et al.*, 2019 ▸), including anti­bacterial (Bouzian *et al.*, 2020 ▸), anti­cancer (Tang *et al.*, 2018 ▸), anti­tubercular (Xu *et al.*, 2017 ▸), anti-COVID19 (Gao *et al.*, 2020 ▸), anti­malarial (Hu *et al.*, 2017 ▸), anti­leishmanial (Palit *et al.*, 2009 ▸) and anti-inflammatory (Pinz *et al.*, 2016 ▸) activities. Furthermore, many studies have shown that quinoline derivatives are good corrosion inhibitors (Douche *et al.* 2020 ▸).
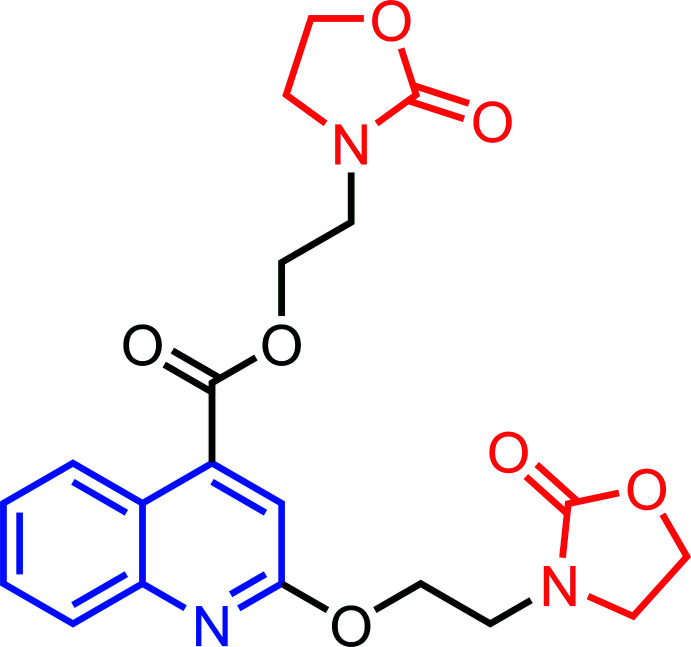


In a continuation of our research work devoted to the syntheses and crystal structures of quinoline derivatives (Bouzian *et al.*, 2019*a* ▸), we report herein the mol­ecular and crystal structures, Hirshfeld surface analysis, DFT and mol­ecular docking investigation of 2-(2-oxo-1,3-oxazolidin-3-yl)ethyl 2-[2-(2-oxo-1,3-oxazolidin-3-yl)eth­oxy]quinoline-4-carboxyl­ate.

## Structural commentary   

**Figure 1:**
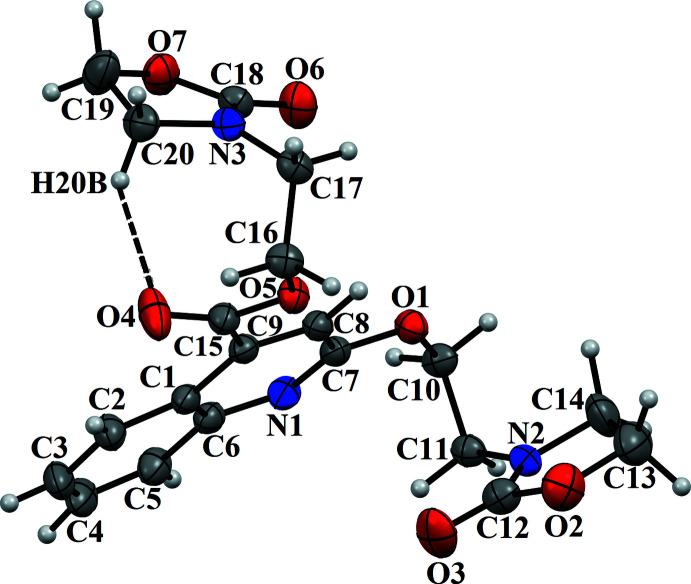
The mol­ecular structure of the title compound, with atom labelling. Displacement ellipsoids are drawn at the 50% probability level. The intra­molecular hydrogen bond is indicated by a dashed line.

In the title mol­ecule (Fig. 1 ▸), the phenyl and pyridine rings of the quinoline system are slightly bent, with a dihedral angle between their mean planes of 3.47 (7)°. The oxazolidine ring (N2/O2/C12–C14) adopts an envelope conformation, with puckering parameters of *Q*(2) = 0.112 (2) Å and φ(2) = 115.3 (10)°. The C14 atom is at the envelope flap position, and it deviates from the least-square plane through the remaining four atoms by 0.070 (2) Å. The other oxazolidine ring (N3/O7/C18–C20) has a twisted conformation along the C20—C19 bond, with puckering parameters *Q*(2) = 0.1732 (18) Å and φ(2) = 299.7 (6)°. The dihedral angles between the mean planes of the oxazolidine rings and the quinoline ring systems are 38.04 (9)° for (N2/O2/C12–C14) and 57.34 (8)° for (N3/O7/C18–C20). The mol­ecular conformation is stabilized by an intra­molecular C20—H20*B*⋯O4 contact (Fig. 1 ▸, Table 1 ▸), producing an *S*(8) ring motif.

## Supra­molecular features   

**Figure 2:**
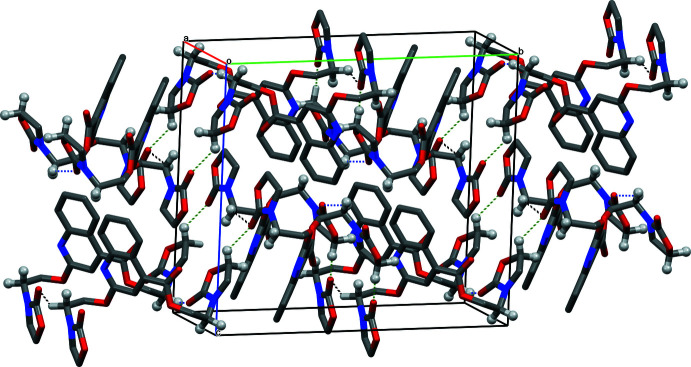
The crystal packing of the title compound, with C11—H11*A*⋯O3^i^, C17—H17*A*⋯O6^ii^ and C19—H19*B*⋯O3^iii^ inter­actions shown as black, blue and green dashed lines, respectively.

In the crystal, C11—H11*A*⋯O3^i^ and C17—H17*A*⋯O6^ii^ hydrogen bonds between methyl­ene groups and carbonyl O atoms as well as C19—H19*B*⋯O3^iii^ hydrogen bonds lead to the formation of corrugated layers extending parallel to (001) (Fig. 2 ▸, Table 1 ▸). Notable C—H⋯π and π–π inter­actions are not observed.

## Database survey   

A search of the Cambridge Structural Database (CSD, version 5.40, update of August 2019; Groom *et al.*, 2016 ▸) using ethyl quinoline-4-carboxyl­ate as the main skeleton revealed the presence of ten structures with different substituents on the quinoline ring. The three structures most similar to the title compound are ethyl 2-(2,4,5-tri­meth­oxy­phen­yl)quinoline-4-carboxyl­ate (OJAGUD; Shrungesh Kumar *et al.*, 2015 ▸), ethyl 2-(3,5-di­fluoro­phen­yl)quinoline-4-carboxyl­ate (UHUHAI; Sunitha *et al.*, 2015 ▸) and ethyl 6-chloro-2-eth­oxy­quinoline-4-carboxyl­ate (XOFGAD; Bouzian *et al.*, 2019*b* ▸). In OJAGUD, the dihedral angle between the quinoline ring system (r.m.s. deviation = 0.028 Å) and the tri­meth­oxy­benzene ring is 43.38 (5)°. A short intra­molecular C—H⋯O contact closes an *S*(6) ring. In the crystal structure, inversion dimers linked by pairs of weak C—H⋯O inter­actions generate *R*_2_^2^(6) loops. In UHUHAI, the two rings of the quinoline system have a dihedral angle of 2.28 (8)° between their mean planes. The plane of the attached benzene ring is inclined to the plane of the quinoline system by 7.65 (7)°. There is a short intra­molecular C—H⋯O contact involving the carbonyl group. In XOFGAD, the mol­ecule is essentially planar with the mean plane of the ethyl acetate group making a dihedral angle of 5.02 (3)° with the ethyl 6-chloro-2-eth­oxy­quinoline mean plane. There is an intra­molecular C— H⋯O hydrogen bond forming an *S*(6) graph-set motif. Weak inter­molecular π–π inter­actions are observed in this crystal structure.

## Hirshfeld surface analysis   

**Figure 3:**
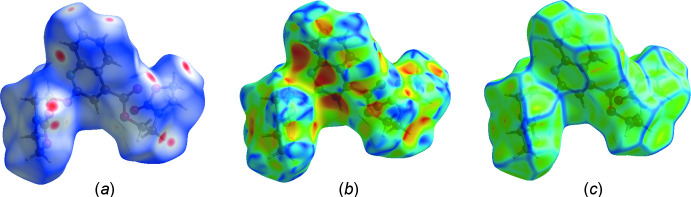
(*a*) *d*_norm_ mapped on the Hirshfeld surface to visualize the inter­molecular inter­actions, (*b*) shape-index map of the title compound and (*c*) curvedness map of the title compound using a range from −4 to 4 Å.

**Figure 4:**
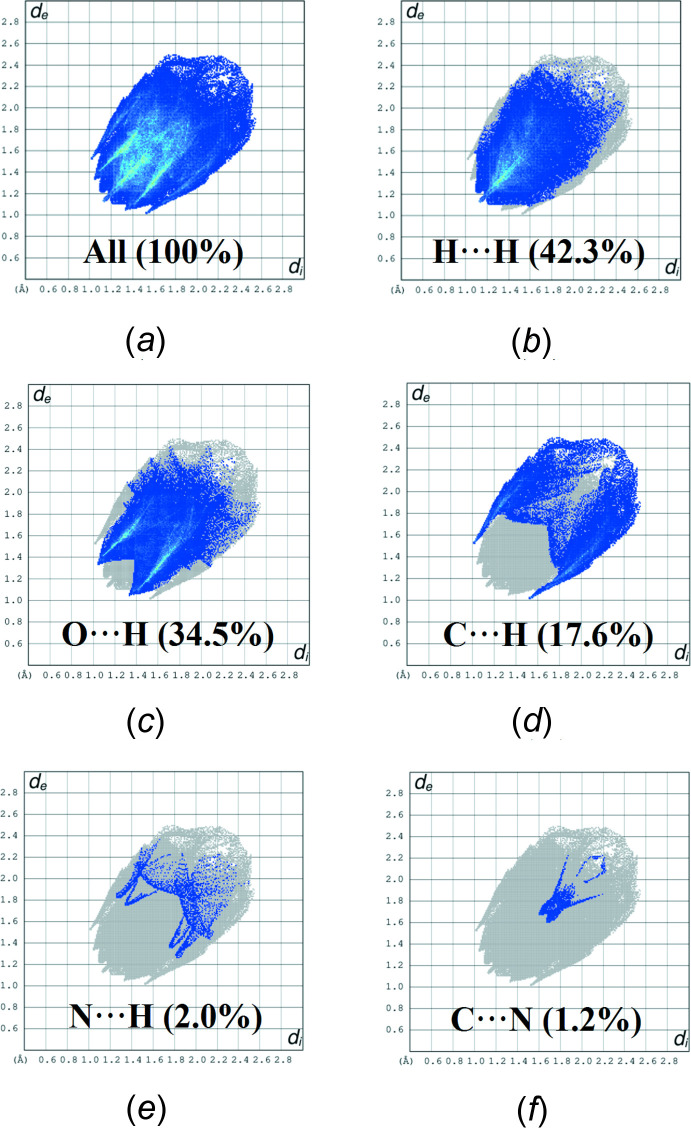
The full two-dimensional fingerprint plots for the title compound, showing (*a*) all inter­actions, and delineated into (*b*) H⋯H, (*c*) H⋯O/O⋯H, (*d*) H⋯C/ C⋯H, (*e*) H⋯N/N⋯H and (*f*) C⋯N/N⋯C inter­actions.

Hirshfeld surface analysis was used to qu­antify the inter­molecular contacts of the title compound, using *Crystal Explorer* (Turner *et al.*, 2017 ▸). The Hirshfeld surface was generated with a standard (high) surface resolution and with the three-dimensional *d*_norm_ surface plotted over a fixed colour scale of −0.1538 (red) to 1.1337 (blue) a.u. (Fig. 3 ▸ *a*). The pale-red spots symbolize short contacts and negative *d*_norm_ values on the surface and correspond to the C—H⋯O inter­actions (Table 1 ▸). The shape-index map of the title mol­ecule was generated in the range −1 to 1 Å (Fig. 3 ▸ *b*). The convex blue regions symbolize hydrogen-donor groups and the concave red regions hydrogen-acceptor groups. The absence of adjacent red and blue triangles in the shape-index map, which generally indicate π–π inter­actions, reveals that this kind of inter­action is not present in the title compound. The curvedness map was generated in the range −4.0 to 4.0 Å (Fig. 3 ▸ *c*). It shows large regions of green with a relatively flat (*i.e.* planar) surface area while the blue regions demonstrate areas of curvature. The overall two-dimensional fingerprint plot is illustrated in Fig. 4 ▸ *a*, with those delineated into H⋯H, H⋯O/O⋯H, H⋯C/ C⋯H, H⋯N/N⋯H and C⋯N/N⋯C contacts associated with their relative contributions to the Hirshfeld surface given in Fig. 4 ▸ *a*–*f*, respectively. The most important inter­molecular inter­actions are H⋯H, contributing 42.3% to the overall crystal packing. H⋯O/O⋯H contacts arising from inter­molecular C—H⋯O hydrogen bonding (Table 1 ▸) make a 34.5% contribution to the Hirshfeld surface and are represented by a pair of sharp spikes in the region *d*_e_ + *d*_i_ ∼2.35 Å (Fig. 4 ▸ *c*). The pair of wings in the fingerprint plot delineated into H⋯C/ C⋯H contacts (17.6% contribution to the Hirshfeld surface) have a nearly symmetrical distribution of points, with the tips at *d*_e_ + *d*_i_ ∼2.54 Å. The contributions of the other contacts to the Hirshfeld surface are negligible, *i.e*. H⋯N/N⋯H of 2.0% and C⋯N/N⋯C of 1.2%.

## Frontier mol­ecular orbital analyses   

**Figure 5:**
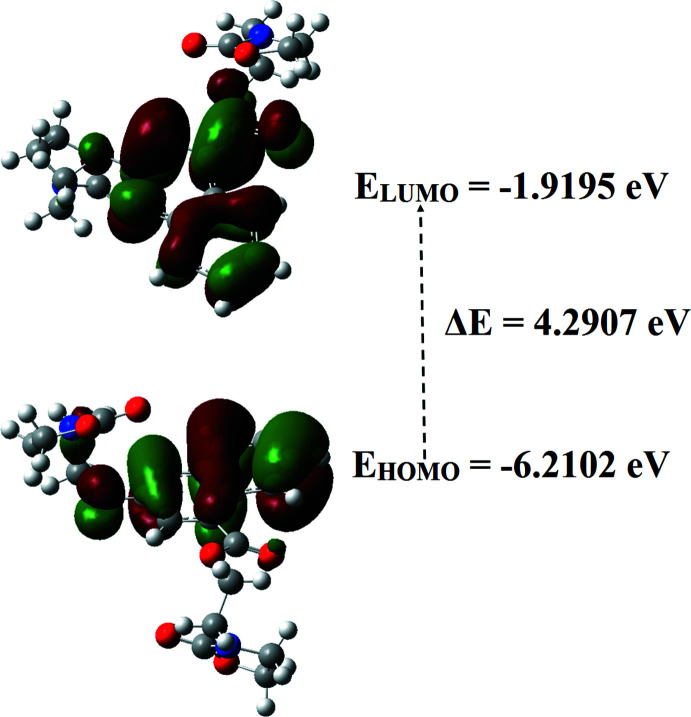
Mol­ecular orbital energy levels of the title compound.

The energy levels for the title compound were computed on basis of density functional theory (DFT) using the standard B3LYP functional and 6–311G++ (d,p) basis-set calculations (Becke, 1993 ▸) as implemented in *GAUSSIAN 09* (Frisch *et al.*, 2009 ▸). The HOMO (highest occupied mol­ecular orbital) acts as an electron donor and the LUMO (lowest occupied mol­ecular orbital) as an electron acceptor. The energy levels, energy gaps, the ionization potential (IP), electron affinity (EA), the chemical potential (μ), the electronegativity (χ), chemical hardness (η), chemical softness (σ), and the electrophilicity index (ω) are given in Table 2 ▸. The electron transition from the HOMO to the LUMO energy level is shown in Fig. 5 ▸. If a mol­ecule has a large HOMO–LUMO energy gap, it can be considered as hard with a low polarizability and a low chemical reactivity. Based on the numerical values collated in Table 2 ▸, the title compound can be classified as a hard material with a HOMO–LUMO energy gap of 4.2907 eV.

## Mol­ecular electrostatic potentials   

**Figure 6:**
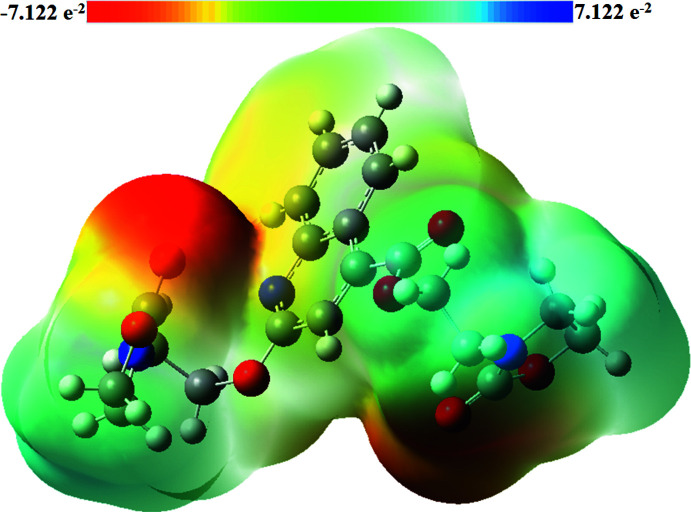
Theoretical mol­ecular electrostatic potential surface calculated at the DFT/B3LYP/6–311 G++ (d,p) basis set level.

The mol­ecular electrostatic potential (MEP) map (Fig. 6 ▸) was calculated at the B3LYP/6-311G++ (d,p) level of theory. In the MEP diagram, the mol­ecular electrostatic potential is in the range −7.122 e^−2^ to 7.122 e^−2^, and the different electrostatic potentials at the surface of the mol­ecule are represented by different colours. Electrostatic potentials increase in the order of red < yellow < green < blue, and red indicates the electron-rich region and blue indicates the electron-deficient region. As shown in Fig. 6 ▸, the carbonyl groups are surrounded by negative charges, indicating some possible nucleophilic attack sites. In addition, the positive charge regions are located on the H atoms.

## Mol­ecular docking study   

**Figure 7:**
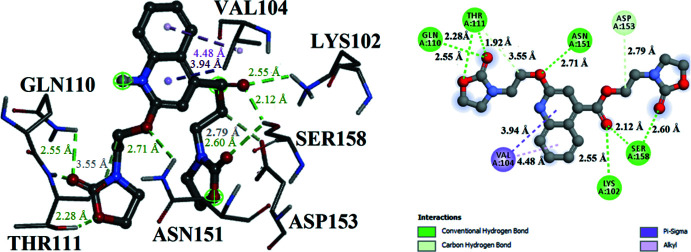
Three- and two-dimensional visuals of the inter­molecular inter­actions for the best binding pose of the title compound docking with the residues of macromolecule 6LU7.

A mol­ecular docking study was performed to determine possible inter­molecular inter­actions between the COVID-19 main protease (PDB ID: 6LU7) and the title mol­ecule. The crystal structure of COVID-19 main protease in a complex with an inhibitor N3 was taken from the RSCB Protein Data Bank (PDB ID: 6LU7; Jin *et al.*, 2020 ▸). The mol­ecular docking study was carried out using *PyRx AutoDock Vina Wizard*. The inter­molecular inter­actions between the title compound and the target protein were visualized by using the *Discovery Studio* 2020 Client program (Biovia, 2017 ▸). The active sites of this target protein are residues LYS102, VAL104, GLN110, THR111, ASN151, ASP153 and SER158. Grid box sizes were determined as 25 × 25 × 25 Å^3^ and *x*, *y*, *z* centers: −10.865636, 12.146782, and 68.902236. The binding affinity energy values and their r.m.s.d. (root-mean-square deviation) values for nine different poses of the ligand docked onto receptor 6LU7 are listed in Table 3 ▸. According to the affinity binding energies, the best binding was determined with −6.3 (kcal mol^−1^) energy and nine active hydrogen-bonding sites. The 2D and 3D visuals of the inter­molecular inter­actions for the best binding pose of the title compound docked into macromolecule 6LU7 can be seen in Fig. 7 ▸. Table 4 ▸ lists details of inter­molecular hydrogen-bonding inter­actions between the title mol­ecule and the macromolecule 6LU7. Additionally in Fig. 7 ▸, π–σ and alkyl inter­actions and their bonding distances are shown. The title mol­ecule appears to be a good agent because of its affinity and ability to adhere to the active sites of the protein.

## Synthesis and crystallization   

A solution of 0.8 g (4.23 mmol) of 2-oxo-1,2-di­hydro­quinoline-4-carb­oxy­lic acid in 30 ml of DMF was mixed with 1.5 g (8.46 mmol) bis­(2-chloro­eth­yl)amine hydro­chloride, 2.33 g (16,92 mmol) K_2_CO_3_ and 0.13 g (0.423 mmol) tetra-*n*-butyl­ammonium bromide (TBAB). The reaction mixture was stirred at 363 K for 9 h in DMF. After removal of formed salts by filtration, DMF was evaporated under reduced pressure, and the residue obtained was dissolved in di­chloro­methane. The organic phase was dried over Na_2_SO_4_ and then concentrated *in vacuo*. The resulting mixture was chromatographed on a silica gel column [eluent: ethyl acetate/hexane (2/8 *v*/*v*)]. Colourless single crystals of the title compound were obtained by slow evaporation of an ethanol solution.

## Refinement   

Crystal data, data collection and structure refinement details are summarized in Table 5 ▸. Hydrogen atoms were discernible from difference Fourier maps and were refined freely.

## Supplementary Material

Crystal structure: contains datablock(s) I. DOI: 10.1107/S2056989020015960/wm5588sup1.cif

Structure factors: contains datablock(s) I. DOI: 10.1107/S2056989020015960/wm5588Isup2.hkl

Supporting information file. DOI: 10.1107/S2056989020015960/wm5588Isup3.cml

CCDC reference: 2048734

Additional supporting information:  crystallographic information; 3D view; checkCIF report

## Figures and Tables

**Table 1:** Hydrogen-bond geometry (Å, °)

*D*—H⋯*A*	*D*—H	H⋯*A*	*D*⋯*A*	*D*—H⋯*A*
C2—H2⋯O4	0.969 (19)	2.335 (18)	2.919 (2)	118.1 (13)
C20—H20*B*⋯O4	0.98 (2)	2.52 (2)	3.275 (2)	133.7 (15)
C11—H11*A*⋯O3^i^	1.026 (19)	2.486 (19)	3.262 (2)	131.8 (13)
C17—H17*A*⋯O6^ii^	0.98 (2)	2.53 (2)	3.219 (2)	127.0 (15)
C19—H19*B*⋯O3^iii^	0.90 (3)	2.51 (3)	3.157 (2)	129 (2)

Symmetry codes: (i) 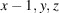; (ii) 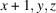; (iii) 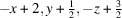.

**Table 2:** Calculated frontier mol­ecular orbital energies (eV)

FMO	Energy
*E*(HOMO)	−6.2102
*E*(LUMO)	−1.9195
Energy gap (Δ*E*)	4.2907
Ionization potential (*IP*)	6.2102
Electron affinity (*EA*)	1.9195
Chemical potential (μ)	–4.0649
Electronegativity (χ)	4.0649
Chemical hardness (η)	2.1454
Chemical softness (σ)	0.2331
Electrophilicity index (ω)	3.8509

**Table 3:** The list of binding affinities and r.m.s.d. values of different sites in protein (6LU7) of the title compound

Ligand	Affinity (kcal mol^−1^)	r.m.s.d./ub	r.m.s.d./Ib
6LU7_ligand	−6.3	0.0	0.0
6LU7_ligand	−6.1	4.8	2.164
6LU7_ligand	−5.8	20.521	17.722
6LU7_ligand	−5.7	20.874	18.477
6LU7_ligand	−5.7	20.28	17.737
6LU7_ligand	−5.6	21.789	19.301
6LU7_ligand	−5.6	20.948	18.265
6LU7_ligand	−5.5	21.63	19.349
6LU7_ligand	−5.4	21.972	19.381

**Table 4:** The inter­molecular hydrogen-bonding inter­actions with the distances (Å) between the title mol­ecule and the macromolecule 6LU7

Residue group	Ligand group	Distance	Hydrogen bond
NH_3_ group in LYS102	O atom in ethyl acetate	2.55	Conventional
NH_2_ group in GLN110	O atom in oxazolidine	2.55	Conventional
NH group in THR111	O atom in oxazolidine	1.92	Conventional
OH group in THR111	O atom in oxazolidine	2.28	Conventional
O atom in THR111	CH_2_ group in 1-meth­oxy­propane	3.55	Carbon
NH_2_ group in ASN151	O atom in 1-meth­oxy­propane	2.71	Conventional
O atom in ASP153	CH_2_ group in ethyl acetate	2.79	Carbon
OH group in SER158	O atom in ethyl acetate	2.12	Conventional
OH group in SER158	O atom in oxazolidine	2.60	Conventional

**Table 5:** Experimental details

Crystal data	
Chemical formula	C_20_H_21_N_3_O_7_
*M*_r_	415.40
Crystal system, space group	Monoclinic, *P*2_1_/*c*
Temperature (K)	150
*a*, *b*, *c* (Å)	6.0686 (5), 19.2791 (15), 16.3795 (13)
β (°)	94.185 (4)
*V* (Å^3^)	1911.2 (3)
*Z*	4
Radiation type	Cu *K*α
μ (mm^−1^)	0.93
Crystal size (mm)	0.23 × 0.08 × 0.05

Data collection	
Diffractometer	Bruker D8 VENTURE PHOTON 100 CMOS
Absorption correction	Multi-scan (*SADABS*; Krause *et al.*, 2015 ▸)
No. of measured, independent and observed [*I* > 2σ(*I*)] reflections	14512, 3711, 3072
*R*_int_	0.042
(sin θ/λ)_max_ (Å^−1^)	0.618

Refinement	
*R*[*F* ^2^ > 2σ(*F* ^2^)], *wR*(*F* ^2^), *S*	0.038, 0.094, 1.05
No. of reflections	3711
No. of parameters	356
H-atom treatment	All H-atom parameters refined
Δρ_max_, Δρ_min_ (e Å^−3^)	0.22, −0.19

Computer programs: *APEX3* and *SAINT* (Bruker, 2016 ▸), *SHELXT* (Sheldrick, 2015*a* ▸), *SHELXL2018/1* (Sheldrick, 2015*b* ▸), *Mercury* (Macrae *et al.*, 2020 ▸), *PLATON* (Spek, 2020 ▸) and *publCIF* (Westrip, 2010 ▸).

## supplementary crystallographic information

### Crystal data

**Table:**

C_20_H_21_N_3_O_7_	*F*(000) = 872
*M**_r_* = 415.40	*D*_x_ = 1.444 Mg m^−^^3^
Monoclinic, *P*2_1_/*c*	Cu *K*α radiation, λ = 1.54178 Å
*a* = 6.0686 (5) Å	Cell parameters from 9277 reflections
*b* = 19.2791 (15) Å	θ = 3.6–72.3°
*c* = 16.3795 (13) Å	µ = 0.93 mm^−^^1^
β = 94.185 (4)°	*T* = 150 K
*V* = 1911.2 (3) Å^3^	Column, colourless
*Z* = 4	0.23 × 0.08 × 0.05 mm

### Data collection

**Table:**

Bruker D8 VENTURE PHOTON 100 CMOS diffractometer	3711 independent reflections
Radiation source: INCOATEC IµS micro–focus source	3072 reflections with *I* > 2σ(*I*)
Detector resolution: 10.4167 pixels mm^-1^	*R*_int_ = 0.042
ω scans	θ_max_ = 72.3°, θ_min_ = 3.6°
Absorption correction: multi-scan (*SADABS*; Krause *et al.*, 2015)	*h* = −7→6
	*k* = −21→23
14512 measured reflections	*l* = −20→18

### Refinement

**Table:**

Refinement on *F*^2^	Hydrogen site location: difference Fourier map
Least-squares matrix: full	All H-atom parameters refined
*R*[*F*^2^ > 2σ(*F*^2^)] = 0.038	*w* = 1/[σ^2^(*F*_o_^2^) + (0.0346*P*)^2^ + 0.6581*P*] where *P* = (*F*_o_^2^ + 2*F*_c_^2^)/3
*wR*(*F*^2^) = 0.094	(Δ/σ)_max_ < 0.001
*S* = 1.05	Δρ_max_ = 0.22 e Å^−^^3^
3711 reflections	Δρ_min_ = −0.19 e Å^−^^3^
356 parameters	Extinction correction: SHELXL-2018/3 (Sheldrick 2015b), Fc^*^=kFc[1+0.001xFc^2^λ^3^/sin(2θ)]^-1/4^
0 restraints	Extinction coefficient: 0.0026 (2)

### Special details

**Table:**

Geometry. All esds (except the esd in the dihedral angle between two l.s. planes) are estimated using the full covariance matrix. The cell esds are taken into account individually in the estimation of esds in distances, angles and torsion angles; correlations between esds in cell parameters are only used when they are defined by crystal symmetry. An approximate (isotropic) treatment of cell esds is used for estimating esds involving l.s. planes.

### Fractional atomic coordinates and isotropic or equivalent isotropic displacement parameters (Å^2^)

**Table:**

	*x*	*y*	*z*	*U*_iso_*/*U*_eq_	
O1	0.37759 (18)	0.21379 (5)	0.57549 (6)	0.0312 (3)	
O2	0.7629 (2)	0.08767 (7)	0.44369 (8)	0.0493 (3)	
O3	0.7633 (2)	0.07204 (8)	0.57987 (8)	0.0506 (3)	
O4	1.1362 (2)	0.34395 (7)	0.75827 (7)	0.0530 (4)	
O5	1.07647 (17)	0.32859 (5)	0.62287 (6)	0.0297 (2)	
O6	0.68920 (19)	0.45136 (7)	0.59441 (7)	0.0426 (3)	
O7	0.76696 (19)	0.50976 (7)	0.71190 (7)	0.0410 (3)	
N1	0.4550 (2)	0.18848 (6)	0.71212 (8)	0.0307 (3)	
N2	0.4425 (2)	0.08645 (7)	0.49889 (8)	0.0292 (3)	
N3	1.0450 (2)	0.48184 (6)	0.63804 (8)	0.0284 (3)	
C1	0.7992 (3)	0.23409 (7)	0.78094 (9)	0.0275 (3)	
C2	0.9438 (3)	0.23392 (9)	0.85269 (10)	0.0355 (4)	
C3	0.8913 (3)	0.19732 (9)	0.92045 (10)	0.0410 (4)	
C4	0.6930 (3)	0.16004 (9)	0.92016 (10)	0.0409 (4)	
C5	0.5501 (3)	0.15931 (9)	0.85188 (10)	0.0367 (4)	
C6	0.6007 (3)	0.19519 (8)	0.78029 (9)	0.0295 (3)	
C7	0.5068 (2)	0.21938 (7)	0.64584 (9)	0.0273 (3)	
C8	0.6967 (3)	0.26216 (7)	0.63962 (9)	0.0270 (3)	
C9	0.8405 (3)	0.27009 (7)	0.70638 (9)	0.0263 (3)	
C10	0.2011 (3)	0.16344 (8)	0.57237 (10)	0.0316 (3)	
C11	0.2925 (3)	0.09066 (8)	0.56362 (10)	0.0296 (3)	
C12	0.6613 (3)	0.08141 (8)	0.51449 (10)	0.0343 (4)	
C13	0.5965 (4)	0.09582 (12)	0.37669 (12)	0.0521 (5)	
C14	0.3811 (4)	0.10463 (13)	0.41522 (11)	0.0503 (5)	
C15	1.0341 (3)	0.31748 (8)	0.70093 (9)	0.0296 (3)	
C16	1.2533 (3)	0.37628 (8)	0.60659 (10)	0.0307 (3)	
C17	1.1579 (3)	0.44476 (8)	0.57658 (10)	0.0305 (3)	
C18	0.8247 (2)	0.47778 (8)	0.64262 (9)	0.0293 (3)	
C19	0.9624 (4)	0.54073 (12)	0.75294 (14)	0.0536 (5)	
C20	1.1552 (3)	0.50923 (9)	0.71305 (11)	0.0368 (4)	
H2	1.087 (3)	0.2562 (10)	0.8534 (10)	0.035 (5)*	
H3	0.995 (4)	0.1973 (11)	0.9668 (13)	0.051 (6)*	
H4	0.662 (3)	0.1323 (10)	0.9671 (12)	0.044 (5)*	
H5	0.409 (3)	0.1320 (10)	0.8502 (12)	0.047 (5)*	
H8	0.718 (3)	0.2863 (9)	0.5880 (11)	0.031 (4)*	
H10A	0.104 (3)	0.1775 (9)	0.5222 (11)	0.033 (4)*	
H10B	0.116 (3)	0.1663 (9)	0.6224 (11)	0.034 (4)*	
H11A	0.158 (3)	0.0587 (9)	0.5522 (11)	0.037 (5)*	
H11B	0.378 (3)	0.0759 (9)	0.6148 (11)	0.034 (5)*	
H13A	0.593 (5)	0.0502 (15)	0.3426 (16)	0.084 (8)*	
H13B	0.637 (5)	0.1344 (15)	0.3451 (17)	0.087 (9)*	
H14A	0.267 (5)	0.0739 (14)	0.3906 (16)	0.075 (8)*	
H14B	0.331 (5)	0.1581 (17)	0.4123 (17)	0.099 (10)*	
H16A	1.355 (3)	0.3820 (9)	0.6562 (11)	0.031 (4)*	
H16B	1.332 (3)	0.3535 (9)	0.5624 (11)	0.035 (5)*	
H17A	1.281 (3)	0.4738 (10)	0.5615 (12)	0.044 (5)*	
H17B	1.047 (3)	0.4375 (9)	0.5297 (11)	0.029 (4)*	
H19A	0.942 (5)	0.5932 (16)	0.7433 (17)	0.092 (9)*	
H19B	0.962 (4)	0.5331 (13)	0.8072 (17)	0.073 (8)*	
H20A	1.268 (4)	0.5440 (13)	0.6998 (15)	0.068 (7)*	
H20B	1.224 (3)	0.4711 (11)	0.7450 (12)	0.048 (5)*	

### Atomic displacement parameters (Å^2^)

**Table:**

	*U*^11^	*U*^22^	*U*^33^	*U*^12^	*U*^13^	*U*^23^
O1	0.0313 (6)	0.0295 (5)	0.0318 (6)	−0.0034 (4)	−0.0036 (4)	0.0011 (4)
O2	0.0420 (7)	0.0586 (8)	0.0491 (7)	−0.0029 (6)	0.0164 (6)	−0.0012 (6)
O3	0.0275 (6)	0.0764 (10)	0.0462 (7)	0.0044 (6)	−0.0090 (5)	−0.0006 (7)
O4	0.0718 (9)	0.0576 (8)	0.0280 (6)	−0.0357 (7)	−0.0071 (6)	0.0048 (6)
O5	0.0317 (6)	0.0320 (5)	0.0259 (5)	−0.0065 (4)	0.0062 (4)	0.0000 (4)
O6	0.0255 (6)	0.0581 (8)	0.0438 (7)	−0.0046 (5)	−0.0020 (5)	−0.0064 (6)
O7	0.0361 (7)	0.0504 (7)	0.0372 (6)	0.0060 (5)	0.0066 (5)	−0.0059 (5)
N1	0.0342 (7)	0.0287 (6)	0.0296 (7)	−0.0023 (5)	0.0055 (5)	−0.0018 (5)
N2	0.0276 (7)	0.0309 (7)	0.0284 (6)	0.0029 (5)	−0.0026 (5)	0.0007 (5)
N3	0.0232 (6)	0.0301 (6)	0.0316 (7)	−0.0013 (5)	−0.0009 (5)	−0.0002 (5)
C1	0.0368 (8)	0.0227 (7)	0.0233 (7)	−0.0003 (6)	0.0048 (6)	−0.0011 (5)
C2	0.0455 (10)	0.0336 (8)	0.0269 (8)	−0.0060 (7)	0.0000 (7)	0.0012 (6)
C3	0.0607 (12)	0.0382 (9)	0.0234 (8)	−0.0038 (8)	−0.0020 (7)	0.0022 (7)
C4	0.0638 (12)	0.0347 (8)	0.0252 (8)	−0.0061 (8)	0.0103 (8)	0.0022 (7)
C5	0.0480 (10)	0.0327 (8)	0.0307 (8)	−0.0067 (7)	0.0118 (7)	−0.0007 (6)
C6	0.0370 (9)	0.0257 (7)	0.0266 (7)	0.0001 (6)	0.0071 (6)	−0.0018 (6)
C7	0.0288 (8)	0.0244 (7)	0.0284 (7)	0.0020 (6)	0.0005 (6)	−0.0017 (6)
C8	0.0315 (8)	0.0245 (7)	0.0251 (7)	0.0019 (6)	0.0035 (6)	0.0012 (6)
C9	0.0326 (8)	0.0218 (7)	0.0250 (7)	−0.0002 (6)	0.0047 (6)	−0.0004 (5)
C10	0.0252 (8)	0.0298 (8)	0.0389 (9)	−0.0008 (6)	−0.0021 (7)	−0.0017 (7)
C11	0.0261 (8)	0.0292 (8)	0.0331 (8)	−0.0003 (6)	−0.0001 (6)	0.0016 (6)
C12	0.0285 (8)	0.0347 (8)	0.0399 (9)	−0.0005 (6)	0.0029 (7)	−0.0017 (7)
C13	0.0700 (14)	0.0514 (12)	0.0361 (10)	−0.0016 (10)	0.0106 (9)	0.0036 (9)
C14	0.0582 (13)	0.0632 (13)	0.0283 (9)	0.0099 (10)	−0.0041 (8)	0.0066 (8)
C15	0.0362 (9)	0.0264 (7)	0.0262 (7)	−0.0026 (6)	0.0029 (6)	0.0010 (6)
C16	0.0258 (8)	0.0319 (8)	0.0351 (8)	−0.0035 (6)	0.0071 (6)	0.0039 (6)
C17	0.0263 (8)	0.0339 (8)	0.0321 (8)	−0.0012 (6)	0.0062 (6)	0.0055 (6)
C18	0.0249 (8)	0.0328 (8)	0.0303 (8)	0.0008 (6)	0.0026 (6)	0.0033 (6)
C19	0.0502 (12)	0.0574 (13)	0.0509 (12)	0.0130 (10)	−0.0111 (9)	−0.0218 (10)
C20	0.0340 (9)	0.0346 (9)	0.0406 (9)	−0.0054 (7)	−0.0059 (7)	−0.0023 (7)

### Geometric parameters (Å, º)

**Table:**

O1—C7	1.3499 (18)	C4—H4	0.97 (2)
O1—C10	1.4437 (19)	C5—C6	1.414 (2)
O2—C12	1.358 (2)	C5—H5	1.00 (2)
O2—C13	1.445 (3)	C7—C8	1.427 (2)
O3—C12	1.211 (2)	C8—C9	1.357 (2)
O4—C15	1.2006 (19)	C8—H8	0.982 (18)
O5—C15	1.3396 (18)	C9—C15	1.496 (2)
O5—C16	1.4526 (18)	C10—C11	1.519 (2)
O6—C18	1.2100 (19)	C10—H10A	1.013 (18)
O7—C18	1.3596 (19)	C10—H10B	1.001 (19)
O7—C19	1.449 (2)	C11—H11A	1.026 (19)
N1—C7	1.2969 (19)	C11—H11B	0.994 (19)
N1—C6	1.379 (2)	C13—C14	1.502 (3)
N2—C12	1.338 (2)	C13—H13A	1.04 (3)
N2—C14	1.437 (2)	C13—H13B	0.95 (3)
N2—C11	1.449 (2)	C14—H14A	0.98 (3)
N3—C18	1.347 (2)	C14—H14B	1.08 (3)
N3—C17	1.447 (2)	C16—C17	1.509 (2)
N3—C20	1.455 (2)	C16—H16A	0.992 (18)
C1—C2	1.415 (2)	C16—H16B	0.997 (18)
C1—C6	1.418 (2)	C17—H17A	0.98 (2)
C1—C9	1.443 (2)	C17—H17B	0.992 (18)
C2—C3	1.372 (2)	C19—C20	1.509 (3)
C2—H2	0.969 (19)	C19—H19A	1.03 (3)
C3—C4	1.401 (3)	C19—H19B	0.90 (3)
C3—H3	0.95 (2)	C20—H20A	0.99 (3)
C4—C5	1.364 (3)	C20—H20B	0.98 (2)
			
C7—O1—C10	117.84 (12)	C10—C11—H11B	110.9 (11)
C12—O2—C13	108.82 (14)	H11A—C11—H11B	109.8 (15)
C15—O5—C16	118.18 (12)	O3—C12—N2	128.04 (16)
C18—O7—C19	108.80 (13)	O3—C12—O2	122.28 (15)
C7—N1—C6	117.04 (13)	N2—C12—O2	109.68 (14)
C12—N2—C14	112.61 (15)	O2—C13—C14	105.94 (15)
C12—N2—C11	122.16 (13)	O2—C13—H13A	107.6 (15)
C14—N2—C11	123.44 (14)	C14—C13—H13A	109.5 (16)
C18—N3—C17	122.17 (13)	O2—C13—H13B	107.9 (17)
C18—N3—C20	111.80 (13)	C14—C13—H13B	114.0 (18)
C17—N3—C20	123.69 (13)	H13A—C13—H13B	112 (2)
C2—C1—C6	118.71 (14)	N2—C14—C13	101.55 (16)
C2—C1—C9	124.65 (14)	N2—C14—H14A	111.9 (15)
C6—C1—C9	116.63 (14)	C13—C14—H14A	111.7 (16)
C3—C2—C1	120.43 (16)	N2—C14—H14B	109.2 (15)
C3—C2—H2	118.6 (11)	C13—C14—H14B	110.1 (16)
C1—C2—H2	120.8 (10)	H14A—C14—H14B	112 (2)
C2—C3—C4	120.73 (17)	O4—C15—O5	123.69 (14)
C2—C3—H3	117.8 (13)	O4—C15—C9	125.14 (14)
C4—C3—H3	121.5 (13)	O5—C15—C9	111.15 (13)
C5—C4—C3	120.16 (15)	O5—C16—C17	110.03 (13)
C5—C4—H4	119.7 (12)	O5—C16—H16A	110.2 (10)
C3—C4—H4	120.0 (12)	C17—C16—H16A	111.8 (10)
C4—C5—C6	120.75 (16)	O5—C16—H16B	104.6 (11)
C4—C5—H5	121.2 (11)	C17—C16—H16B	110.0 (10)
C6—C5—H5	118.0 (11)	H16A—C16—H16B	109.9 (15)
N1—C6—C5	117.43 (15)	N3—C17—C16	113.29 (13)
N1—C6—C1	123.35 (13)	N3—C17—H17A	107.6 (12)
C5—C6—C1	119.20 (15)	C16—C17—H17A	107.4 (12)
N1—C7—O1	121.12 (14)	N3—C17—H17B	106.3 (10)
N1—C7—C8	124.86 (14)	C16—C17—H17B	110.5 (10)
O1—C7—C8	114.01 (13)	H17A—C17—H17B	111.9 (15)
C9—C8—C7	118.89 (14)	O6—C18—N3	128.17 (15)
C9—C8—H8	121.5 (10)	O6—C18—O7	122.10 (14)
C7—C8—H8	119.6 (10)	N3—C18—O7	109.72 (13)
C8—C9—C1	119.10 (14)	O7—C19—C20	105.53 (14)
C8—C9—C15	118.85 (13)	O7—C19—H19A	104.5 (16)
C1—C9—C15	122.03 (13)	C20—C19—H19A	114.5 (17)
O1—C10—C11	110.45 (12)	O7—C19—H19B	109.2 (17)
O1—C10—H10A	103.6 (10)	C20—C19—H19B	114.8 (17)
C11—C10—H10A	111.5 (10)	H19A—C19—H19B	108 (2)
O1—C10—H10B	111.0 (10)	N3—C20—C19	100.86 (14)
C11—C10—H10B	110.0 (10)	N3—C20—H20A	110.0 (14)
H10A—C10—H10B	110.3 (14)	C19—C20—H20A	113.2 (14)
N2—C11—C10	111.95 (13)	N3—C20—H20B	109.4 (12)
N2—C11—H11A	111.4 (10)	C19—C20—H20B	112.7 (12)
C10—C11—H11A	106.3 (10)	H20A—C20—H20B	110.3 (18)
N2—C11—H11B	106.5 (11)		
			
C6—C1—C2—C3	−0.7 (2)	C11—N2—C12—O3	8.8 (3)
C9—C1—C2—C3	−179.17 (16)	C14—N2—C12—O2	−6.6 (2)
C1—C2—C3—C4	−0.6 (3)	C11—N2—C12—O2	−171.89 (13)
C2—C3—C4—C5	0.5 (3)	C13—O2—C12—O3	177.93 (17)
C3—C4—C5—C6	0.8 (3)	C13—O2—C12—N2	−1.40 (19)
C7—N1—C6—C5	−177.44 (14)	C12—O2—C13—C14	8.3 (2)
C7—N1—C6—C1	0.5 (2)	C12—N2—C14—C13	11.2 (2)
C4—C5—C6—N1	175.98 (15)	C11—N2—C14—C13	176.20 (15)
C4—C5—C6—C1	−2.1 (2)	O2—C13—C14—N2	−11.2 (2)
C2—C1—C6—N1	−175.98 (14)	C16—O5—C15—O4	−1.1 (2)
C9—C1—C6—N1	2.7 (2)	C16—O5—C15—C9	177.08 (12)
C2—C1—C6—C5	2.0 (2)	C8—C9—C15—O4	158.27 (17)
C9—C1—C6—C5	−179.41 (13)	C1—C9—C15—O4	−20.1 (2)
C6—N1—C7—O1	177.84 (13)	C8—C9—C15—O5	−19.91 (19)
C6—N1—C7—C8	−3.2 (2)	C1—C9—C15—O5	161.75 (13)
C10—O1—C7—N1	−10.2 (2)	C15—O5—C16—C17	−103.49 (15)
C10—O1—C7—C8	170.70 (13)	C18—N3—C17—C16	−95.85 (17)
N1—C7—C8—C9	2.4 (2)	C20—N3—C17—C16	65.35 (19)
O1—C7—C8—C9	−178.56 (13)	O5—C16—C17—N3	66.50 (17)
C7—C8—C9—C1	1.1 (2)	C17—N3—C18—O6	−9.0 (3)
C7—C8—C9—C15	−177.26 (13)	C20—N3—C18—O6	−172.23 (16)
C2—C1—C9—C8	175.18 (15)	C17—N3—C18—O7	171.83 (13)
C6—C1—C9—C8	−3.4 (2)	C20—N3—C18—O7	8.61 (18)
C2—C1—C9—C15	−6.5 (2)	C19—O7—C18—O6	−175.41 (17)
C6—C1—C9—C15	174.97 (13)	C19—O7—C18—N3	3.81 (19)
C7—O1—C10—C11	−75.87 (16)	C18—O7—C19—C20	−13.9 (2)
C12—N2—C11—C10	105.84 (17)	C18—N3—C20—C19	−16.32 (19)
C14—N2—C11—C10	−57.8 (2)	C17—N3—C20—C19	−179.25 (16)
O1—C10—C11—N2	−48.92 (18)	O7—C19—C20—N3	17.5 (2)
C14—N2—C12—O3	174.08 (19)		

### Hydrogen-bond geometry (Å, º)

**Table:**

*D*—H···*A*	*D*—H	H···*A*	*D*···*A*	*D*—H···*A*
C2—H2···O4	0.969 (19)	2.335 (18)	2.919 (2)	118.1 (13)
C20—H20*B*···O4	0.98 (2)	2.52 (2)	3.275 (2)	133.7 (15)
C11—H11*A*···O3^i^	1.026 (19)	2.486 (19)	3.262 (2)	131.8 (13)
C17—H17*A*···O6^ii^	0.98 (2)	2.53 (2)	3.219 (2)	127.0 (15)
C19—H19*B*···O3^iii^	0.90 (3)	2.51 (3)	3.157 (2)	129 (2)

Symmetry codes: (i) *x*−1, *y*, *z*; (ii) *x*+1, *y*, *z*; (iii) −*x*+2, *y*+1/2, −*z*+3/2.

## References

[bb1] Becke, A. D. (1993). *J. Chem. Phys.* **98**, 5648–5652.

[bb2] Biovia (2017). *Discovery studio visualizer.* Vol. 936. Biovia, San Diego, CA, USA.

[bb3] Bouzian, Y., Faizi, M. S. H., Mague, J. T., Otmani, B. E., Dege, N., Karrouchi, K. & Essassi, E. M. (2019*a*). *Acta Cryst.* E**75**, 980–983. 10.1107/S2056989019007989 PMC6659339 31392008

[bb4] Bouzian, Y., Karrouchi, K., Anouar, E. H., Bouhfid, R., Arshad, S. & Essassi, E. M. (2019*b*). *Acta Cryst.* E**75**, 912–916. 10.1107/S2056989019007473 PMC6658954 31391993

[bb5] Bouzian, Y., Karrouchi, K., Sert, Y., Lai, C.-H., Mahi, L., Ahabchane, N. H., Talbaoui, A., Mague, J. T. & Essassi, E. M. (2020). *J. Mol. Struct.* **1209**, 127940.

[bb6] Bruker (2016). *APEX3* and *SAINT*. Bruker AXS, Inc., Madison, Wisconsin, USA.

[bb7] Chu, X. M., Wang, C., Liu, W., Liang, L. L., Gong, K. K., Zhao, C. Y. & Sun, K. L. (2019). *Eur. J. Med. Chem.* **161**, 101–117. 10.1016/j.ejmech.2018.10.035 30343191

[bb8] Douche, D., Elmsellem, H., Anouar, E. H., Guo, L., Hafez, B., Tüzün, B., El Louzi, A., Bougrin, K., Karrouchi, K. & Himmi, B. (2020). *J. Mol. Liq.* **308**, 113042.

[bb9] Frisch, M. J., Trucks, G. W., Schlegel, H. B., Scuseria, G. E., Robb, M. A., Cheeseman, J. R., Scalmani, G., Barone, V., Mennucci, B., Petersson, G. A., Nakatsuji, H., Caricato, M., Li, X., Hratchian, H. P., Izmaylov, A. F., Bloino, J., Zheng, G., Sonnenberg, J. L., Hada, M., Ehara, M., Toyota, K., Fukuda, R., Hasegawa, J., Ishida, M., Nakajima, T., Honda, Y., Kitao, O., Nakai, H., Vreven, T., Montgomery, J. A. Jr, Peralta, J. E., Ogliaro, F., Bearpark, M., Heyd, J. J., Brothers, E., Kudin, K. N., Staroverov, V. N., Kobayashi, R., Normand, J., Raghavachari, K., Rendell, A., Burant, J. C., Iyengar, S. S., Tomasi, J., Cossi, M., Rega, N., Millam, J. M., Klene, M., Knox, J. E., Cross, J. B., Bakken, V., Adamo, C., Jaramillo, J., Gomperts, R., Stratmann, R. E., Yazyev, O., Austin, A. J., Cammi, R., Pomelli, C., Ochterski, J. W., Martin, R. L., Morokuma, K., Zakrzewski, V. G., Voth, G. A., Salvador, P., Dannenberg, J. J., Dapprich, S., Daniels, A. D., Farkas, O., Foresman, J. B., Ortiz, J. V., Cioslowski, J. & Fox, D. J. (2009). *GAUSSIAN09.* Rev. A.02. Gaussian Inc., Wallingford, CT, USA.

[bb10] Gao, J., Tian, Z. & Yang, X. (2020). *Biosci. Trends*, **14**, 72-73. 10.5582/bst.2020.01047 32074550

[bb11] Groom, C. R., Bruno, I. J., Lightfoot, M. P. & Ward, S. C. (2016). *Acta Cryst.* B**72**, 171–179. 10.1107/S2052520616003954 PMC4822653 27048719

[bb12] Hu, Y. Q., Gao, C., Zhang, S., Xu, L., Xu, Z., Feng, L. S., Wu, X. & Zhao, F. (2017). *Eur. J. Med. Chem.* **139**, 22–47. 10.1016/j.ejmech.2017.07.061 28800458

[bb13] Jin, Z., Du, X., Xu, Y., Deng, Y., Liu, M., Zhao, Y., Zhang, B., Li, X., Zhang, L., Peng, C., Duan, Y., Yu, J., Wang, L., Yang, K., Liu, F., Jiang, R., Yang, X., You, T., Liu, X., Yang, X., Bai, F., Liu, H., Liu, X., Guddat, L. W., Xu, W., Xiao, G., Qin, C., Shi, Z., Jiang, H., Rao, Z. & Yang, H. (2020). *Nature*, **582**, 289–293. 10.1038/s41586-020-2223-y 32272481

[bb14] Krause, L., Herbst-Irmer, R., Sheldrick, G. M. & Stalke, D. (2015). *J. Appl. Cryst.* **48**, 3–10. 10.1107/S1600576714022985 PMC4453166 26089746

[bb15] Macrae, C. F., Sovago, I., Cottrell, S. J., Galek, P. T. A., McCabe, P., Pidcock, E., Platings, M., Shields, G. P., Stevens, J. S., Towler, M. & Wood, P. A. (2020). *J. Appl. Cryst.* **53**, 226–235. 10.1107/S1600576719014092 PMC6998782 32047413

[bb16] Palit, P., Paira, P., Hazra, A., Banerjee, S., Gupta, A. D., Dastidar, S. G. & Mondal, N. B. (2009). *Eur. J. Med. Chem.* **44**, 845–853. 10.1016/j.ejmech.2008.04.014 18538452

[bb17] Pinz, M., Reis, A. S., Duarte, V., da Rocha, M. J., Goldani, B. S., Alves, D., Savegnago, L., Luchese, C. & Wilhelm, E. A. (2016). *Eur. J. Pharmacol.* **780**, 122–128. 10.1016/j.ejphar.2016.03.039 27020552

[bb18] Sheldrick, G. M. (2015*a*). *Acta Cryst.* A**71**, 3–8.

[bb19] Sheldrick, G. M. (2015*b*). *Acta Cryst.* C**71**, 3–8.

[bb20] Shrungesh Kumar, T. O., Naveen, S., Kumara, M. N., Mahadevan, K. M. & Lokanath, N. K. (2015). *Acta Cryst.* E**71**, o514–o515. 10.1107/S2056989015011706 PMC4518944 26279938

[bb21] Spek, A. L. (2020). *Acta Cryst.* E**76**, 1–11. 10.1107/S2056989019016244 PMC6944088 31921444

[bb22] Sunitha, V. M., Naveen, S., Manjunath, H. R., Benaka Prasad, S. B., Manivannan, V. & Lokanath, N. K. (2015). *Acta Cryst.* E**71**, o341–o342. 10.1107/S2056989015007677 PMC4420057 25995938

[bb23] Tang, Q. D., Duan, Y. L., Xiong, H. H., Chen, T., Xiao, Z., Wang, L. X., Xiao, Y. Y., Huang, S. M., Xiong, Y., Zhu, W., Gong, P. & Zheng, P. (2018). *Eur. J. Med. Chem.* **158**, 201–213. 10.1016/j.ejmech.2018.08.066 30216852

[bb24] Turner, M. J., McKinnon, J. J., Wolff, S. K., Grimwood, D. J., Spackman, P. R., Jayatilaka, D. & Spackman, M. A. (2017). *CrystalExplorer17.* University of Western Australia. http://hirshfeldsurface.net.

[bb25] Westrip, S. P. (2010). *J. Appl. Cryst.* **43**, 920–925.

[bb26] Xu, Z., Gao, C., Ren, Q. C., Song, X. F., Feng, L. S. & Lv, Z. S. (2017). *Eur. J. Med. Chem.* **139**, 429–440. 10.1016/j.ejmech.2017.07.059 28818767

